# Draft Genome Sequence of *Stenotrophomonas maltophilia* SeITE02, a Gammaproteobacterium Isolated from Selenite-Contaminated Mining Soil

**DOI:** 10.1128/genomeA.00331-14

**Published:** 2014-05-08

**Authors:** Cristina Bertolini, Ronny van Aerle, Silvia Lampis, Karen A. Moore, Konrad Paszkiewicz, Clive S. Butler, Giovanni Vallini, Mark van der Giezen

**Affiliations:** a Department of Biotechnology, University of Verona, Verona, Italy; b Biosciences, University of Exeter, Exeter, United Kingdom

## Abstract

*Stenotrophomonas maltophilia* strain SeITE02 was isolated from the rhizosphere of the selenium-hyperaccumulating legume *Astragalus bisculcatus*. In this report, we provide the 4.56-Mb draft genome sequence of *S. maltophilia* SeITE02, a gammaproteobacterium that can withstand high concentrations of selenite and reduce these to elemental selenium.

## GENOME ANNOUNCEMENT

The microbial contribution to the biogeochemical selenium cycle is of considerable importance and facilitates the mobilization and precipitation of this essential trace element in the biosphere. Bacteria that are capable of the reduction of the oxidized selenium compounds selenate (SeO_4_^2–^) and selenite (SeO_3_^2–^) ultimately result in the precipitation of elemental selenium (Se^0^) (1). These Se nanoparticles are readily observed in either the intracellular or extracellular environment. Unlike the oxidized selenium compounds, Se particles are insoluble and largely nontoxic, thus presenting a microbial strategy for selenium bioremediation. More recently, interest in the properties of the bacterially derived selenium nanoparticles has prompted the exploitation of bacterial biomineralization for the environmentally friendly and cost-efficient fabrication of novel nanomaterials (2). Understanding the enzymatic reactions responsible for the reduction of selenate and selenite is central to advancing these applications. To date, studies of specific enzymes and proteins involved in the Se reductive process have mostly focused on anaerobic selenate-respiring bacteria such as *Thauera selenatis* (3–5). While a number of specific selenate reductases have been reported (5, 6), the details of enzymatic reductions of selenite have remained poorly studied. However, the possible involvement of nitrite reductase, glutathione, and NADH-dependent enzymes has been postulated (7–9). The gammaproteobacterium *Stenotrophomonas maltophilia* is resistant to high concentrations of metals and metalloids and has applications in bioremediation (10, 11). In the present work, we have sequenced the genome of *S. maltophilia* SeITE02 (12) to shed light on the selenite reductive processes.

Here, we present the draft genome sequence of *S. maltophilia* SeITE02. Whole-genome sequencing was performed using an Illumina MiSeq and resulted in 711,594 sequence reads (179 Mb). Reads were quality trimmed using Trimmomatic v0.30 (13) followed by Scythe v0.992beta (https://github.com/vsbuffalo/scythe) and assembled using A5 (14). Scaffolds were annotated using Prokka 1.7 (15) and RAST 4 (16). The genome is 4,557,111 bp in length (GC content, 66.4%), with an average coverage of 34-fold. The draft genome is distributed over 63 scaffolds and contains 4,101 genes (4,032 coding genes and 69 noncoding RNAs [4 rRNAs and 65 tRNAs]). The genome was analyzed to detect known selenate and selenite reductases. The absence of any known selenate reductase (SerABC, SrdBCA, YnfE, and YgfK) (5, 17–19) is consistent with the observation that although *S. maltophilia* SeITE02 tolerates selenate up to the concentration of 60 mM in the growth medium, it does not actively reduce this oxyanion. The NirS-type nitrite reductase, which has been suggested previously to contribute to selenite reduction (7), is also not encoded in the genome. We did, however, identify a glutathione reductase, a thioredoxin reductase, and an NADH:flavin oxidoreductase (OYE family), all of which have been reported to reduce selenite (20).

### Nucleotide sequence accession numbers.

This whole-genome shotgun project has been deposited at DDBJ/ENA/GenBank under the accession no. CBXW000000000. The version described in this paper is the first version, CBXW010000000.
